# PReS-FINAL-2306: Molecular analysis of HLA-DRB1 alleles in Iranian children with juvenile systemic lupus erythematosus

**DOI:** 10.1186/1546-0096-11-S2-P296

**Published:** 2013-12-05

**Authors:** R Shiari, S Farivar, M Dehghan Tezerjani

**Affiliations:** 1 Pediatric Rheumatology, Shahid Beheshti University of Medical Sciences, Mofid Children's Hospital, Tehran, Iran, Islamic Republic Of; 2 Genetics, Shahid Beheshti University, Faculty of Bioscience, Tehran, Iran, Islamic Republic Of

## Introduction

Systemic lupus erythromatosus (SLE) is a complex and systemic autoimmune disease. It is characterized by diverse clinical symptoms, revealing widespread immune-mediated damage. The common clinical features diagnosed in patients with SLE comprise of skin and joint diseases, hematological abnormalities, renal disease and neuropsychiatric complications. Although the etiology of SLE is still unknown, genetic factors are likely to be important in susceptibility to SLE and influence presentation of disease heterogeneity and production of autoantibody in affected subjects.

## Objectives

There are several evidences that Human leukocyte antigens are associated with SLE. Herein, we studied HLA-DRB1 alleles to detect the association of these alleles in Iranian children with juvenile onset SLE.

## Methods

This study consists of 31 children with systemic lupus erythematosus and 56 healthy controls. Genomic DNA was extracted and HLA typing was performed by Polymerase Chain Reaction with Sequence-Specific Primers technique (PCR-SSP).

## Results

HLA- DRB1*01, HLA- DRB1*04, HLA-DRB1*11 and HLA- DRB1*13 were detected to be the most frequent alleles associated with SLE in Iranian children. The frequency of HLA-DRB1*08 was not significantly different in both groups. HLA-DRB1*07 had a higher rate of repetition in the control group than patients with SLE.

## Conclusion

We reached the conclusion that there was a significant difference in the frequency of some alleles between patients and controls which could be related to susceptibility to SLE. This difference may help to predict the onset of lupus in children.

## Disclosure of interest

None declared.

